# Design and Application in Delivery System of Intranasal Antidepressants

**DOI:** 10.3389/fbioe.2020.626882

**Published:** 2020-12-21

**Authors:** Jingying Xu, Jiangang Tao, Jidong Wang

**Affiliations:** ^1^School of Marxism, Yanshan University, Qinhuangdao, China; ^2^Mental Health Service Center, Yanshan University, Qinhuangdao, China; ^3^Applied Chemistry Key Laboratory of Hebei Province, Hebei Key Laboratory of Heavy Metal Deep-Remediation in Water and Resource Reuse, Yanshan University, Qinhuangdao, China

**Keywords:** intranasal route, antidepressants, delivery carrier, design and application, challenges and future perspectives

## Abstract

One of the major reasons why depressed patients fail their treatment course is the existence of the blood-brain barrier (BBB), which prevents drugs from being delivered to the central nervous system (CNS). In recent years, nasal drug delivery has achieved better systemic bioavailability and activity in low doses in antidepressant treatment. In this review, we focused on the latest strategies for delivery carriers (or formation) of intranasal antidepressants. We began this review with an overview of the nasal drug delivery systems, including nasal drug delivery route, absorption mechanism, advantages, and limitations in the nasal drug delivery route. Next, we introduced the development of nasal drug delivery devices, such as powder devices, liquid-based devices, and so on. Finally, intranasal delivery carriers of antidepressants in clinical studies, including nanogels, nanostructured lipid, liposomes nanoparticles, nanoemulsions/microemulsion, were summarized. Moreover, challenges and future perspectives on recent progress of intranasal delivery carriers in antidepressant treatments were discussed.

## Introduction

Depression is a state of low mood and aversion to activity that can affect a person's thoughts, behavior, feelings, physical well-being, and circadian rhythm. Depression has become the leading cause of disability worldwide and is a significant global health burden (Glahn et al., 2012). What worries us is that, although the dozens of antidepressant drugs, which are approved by the Food and Drug Administration (FDA) as shown in Table 1, have demonstrated efficacy in clinical practice, many depressed patients do not respond to the first-line pharmacological treatment and even fail following several pharmacological interventions (Oleary et al., 2015).

**Table 1:** FDA approved antidepressants.

**Classifications**	**Drug name**	**Active ingredients**	**FDA approved year**	**Formations**
Selective serotonin reuptake inhibitors (SSRIs)	PROZAC	Fluoxetine	1987	Capsule
	CELEXA	Citalopram	2000	Tablet
	PAXIL	Paroxetine	1992	Tablet
	VIIBRYD	Vilazodone	2011	Tablet
Serotonin norepinephrine reuptake inhibitors (SNRIs)	EFFEXOR	Venlafaxine	1993	Tablet
	CYMBALTA	Duloxetine	2004	Tablet
	DESVENLAFAXINE	Desvenlafaxine	2013	Tablet
	FETZIMA	Levomilnacipran	2013	Capsule
Tricyclic antidepressants (TCAs)	TOFRANIL	Imipramine	1982	Injection
	PAMELOR	Nortriptyline	1982	Solution
	ELAVIL	Amitriptyline	1982	Tablet
Monoamine oxidase inhibitors (MAOIs)	NARDIL	Phenelzine	1982	Tablet
	PARNATE	Tranylcypromine	1985	Tablet
Other	REMERON	Mirtazapine	1997	Tablet
	WELLBU-TRIN	Bupropion	2002	Tablet

Why is this the case? One of the major reasons patients fail in their treatment course is the existence of the blood-brain barrier (BBB), which is the bottleneck of drug delivery for the central nervous system (CNS). BBB, which is mainly composed of cerebral endothelial cells (CECs) that constitute a selective barrier covering the inner surface of cerebral capillaries, is the major site of blood–CNS exchange, maintaining the homeostasis of the CNS (Bernacki et al., 2008; Abbott et al., 2010). CECs heavily determine the BBB permeability of most circulating compounds. Efflux transmembrane proteins, expressed in endothelial cerebral cells, and particularly those from the ATB-binding cassette family, which mainly include P-glycoprotein (P-gp) and breast-cancer-resistant protein (BCRP), are included in the BBB (Bicker et al., 2014; Tang et al., 2017). The ability to cross this biological barrier has been the determining characteristic for the effectiveness of CNS drugs. To date, there are three strategies used to deliver therapeutics across the barrier of the BBB (Sun et al., 2017; Aderibigbe and Naki, 2019; Saeedi et al., 2019; Zhang M. et al., 2019; Shi et al., 2020): (1) by other routes of drug delivery including drug-encapsulated wafers inserted in the tumor cavity, facial intradermal injection, and drugs administered *via* the nasal cavity; (2) by interrupting the BBB using surfactants and hyperosmotic agents, cell-penetrating peptides, or magnetic nanoparticle-induced hyperthermia; and (3) by endogenous transporters and receptors for enhanced neural non-invasive penetration of drugs. Although antidepressant drugs have recently been identified as substrates, inhibitors, and inductors of P-gp (Obrien et al., 2012), an effective therapy with antidepressant drugs depends on drug concentrations and bioactivity, but conventional oral and parenteral therapies are limited due to the difficulty in crossing the BBB (Vitorino et al., 2019). Nasal mucosa as a potential administration route has achieved faster and higher levels of drug absorption because more compounds could permeate and administrate due to the high permeability, high vasculature, low enzymatic environment of nasal cavity, and avoidance of the hepatic first pass metabolism (Jadhav et al., 2007; Alagusundaram et al., 2010). In recent years, nasal drug delivery has achieved better systemic bioavailability and activity in low doses because the nasal route avoids the hepatic first pass elimination associated with the oral delivery. Intranasal drug delivery proposed a reliable method to bypass the BBB. Due to a direct connection between the brain and the nasal cavity, intranasal administration is the preferred route from the outside environment. Moreover, intranasal formulations have been developed to degrade enzymatic ion and improve the pharmacological effects. More and more pharmaceutical scientists and clinicians have given increasing attention to drug delivery via the nasal route, as shown in Table 2 (Kanojia et al., 2017; Ambrus et al., 2020; Shetty et al., 2020).

**Table 2:** Preparation materials for several common delivery carriers of intranasal antidepressants.

**Classifications**	**Common materials**	**Example**	**References**
Nanogels	Lutrol F127	Venlafaxine	Bhandwalkar and Avachat, 2013
	Chitosan- glycerophosphate	Doxepin	Naik and Nair, 2014
	Chitosan-Pluronic and HPMC	Tramadol HCl	Kaur et al., 2014
Nanostructured lipid	Solid lipid (glyceryl monostearate) and liquid lipid (capryol PGMC)	DLX	Alam et al., 2014
Liposomes	Mixture of egg phosphatidylcholine (EPC) and cholesterol (chol)	Piperine	Priprem et al., 2011
Nanoparticles	Chitosan nanoparticles	Venlafaxine	Haque et al., 2012
	Alginate chitosan nanoparticles (VLF AG-NPs)	Venlafaxine	Haque et al., 2014
	PLGA-chitosan nanoparticles	Desvenlafaxine	Tong et al., 2017
Nanoemulsions /microemulsion	Capmul MCM, Solutol HS 15, and propylene glycol	Paroxetine	Pandey et al., 2016
	Capmul MCM (O-7% w/w) –TRIN	Mirtazapine	Thakkar et al., 2013

**Scheme 1:**
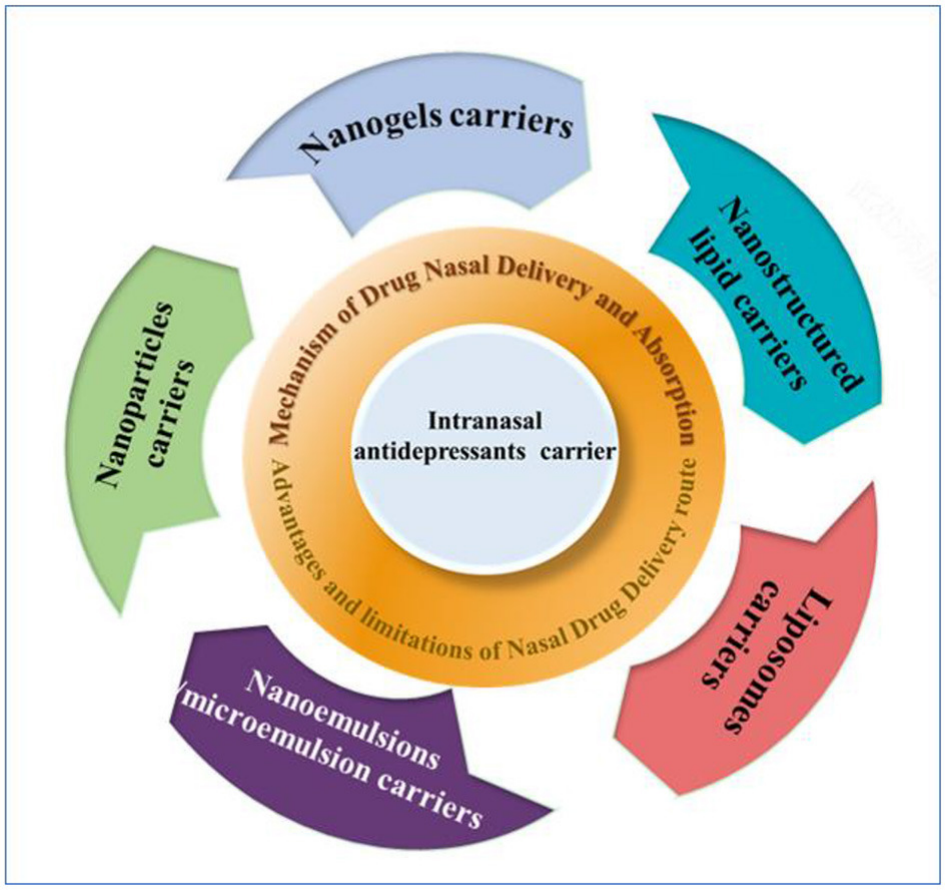
Schematic illustration of the design and application in delivery carriers of intranasal antidepressants.

In this review, we focused on the latest strategies of delivery carriers of intranasal antidepressants (Scheme 1). We began this review with an overview of the nasal drug delivery systems, including nasal drug delivery route, absorption mechanism, and advantages and limitations in nasal drug delivery route. Next, we introduced the development of nasal drug delivery devices, such as powder devices, liquid-based devices, and so on. Finally, intranasal delivery carriers of antidepressants in clinical studies, including nanogels, nanostructured lipid, liposomes nanoparticles, and nanoemulsions/microemulsion, were summarized. Moreover, challenges and future perspectives on recent progress of intranasal delivery carriers in antidepressant treatment were discussed.

## Nasal Drug Delivery Systems

### Nasal Drug Delivery Route

**Figure 1:**
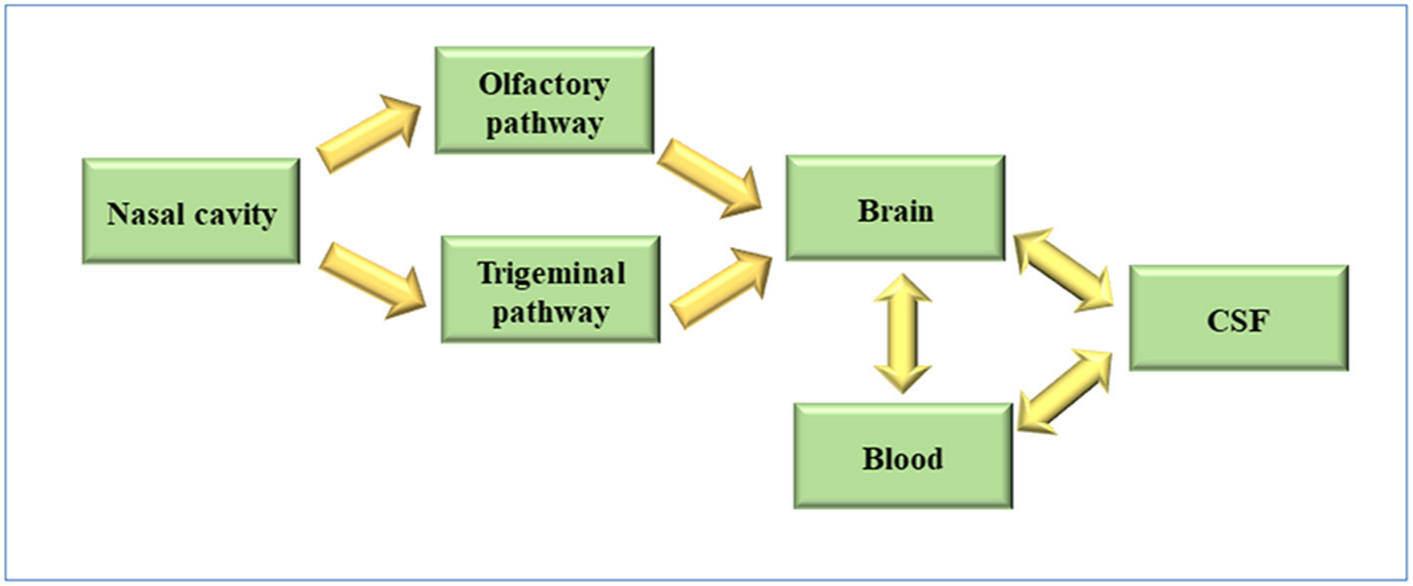
Pathway for brain targeting after intranasal administration.

The nasal route has gained attention as it is a direct non-invasive way to transport drugs to the brain which cannot be transferred *via* the oral route. To date, olfactory and trigeminal nerves have been shown to be safe and effective pathways to deliver therapeutic agents to brain (Figure 1).

The olfactory pathway is composed of the olfactory epithelium, lamina propria, and olfactory bulb. Three types of cells, neuronal cells, progenitor cells, and supporting cells belong to the olfactory epithelium and are connected by tight junctions. An information pathway to the brain is built by neuronal cells which start from the olfactory bulb in the CNS to the olfactory epithelium in the nasal cavity (Leopold, 1988). Due to the constant motion between basal cells and neural cells, the delivery ability of drugs to the brain was enhanced (Caggiano et al., 1994). Lamina propria, which consists of blood vessels, mucus secreting glands, olfactory axons, and a maxillary branch of trigeminal nerve, lies on the nasal epithelium (Brand, 2006; Choi et al., 2020; He et al., 2020). The olfactory bulb is used for direct nasal delivery of drugs for its distribution in different regions of the brain, such as the piriform cortex, amygdala, and hypothalamus (Khan et al., 2017).

**Figure 2:**
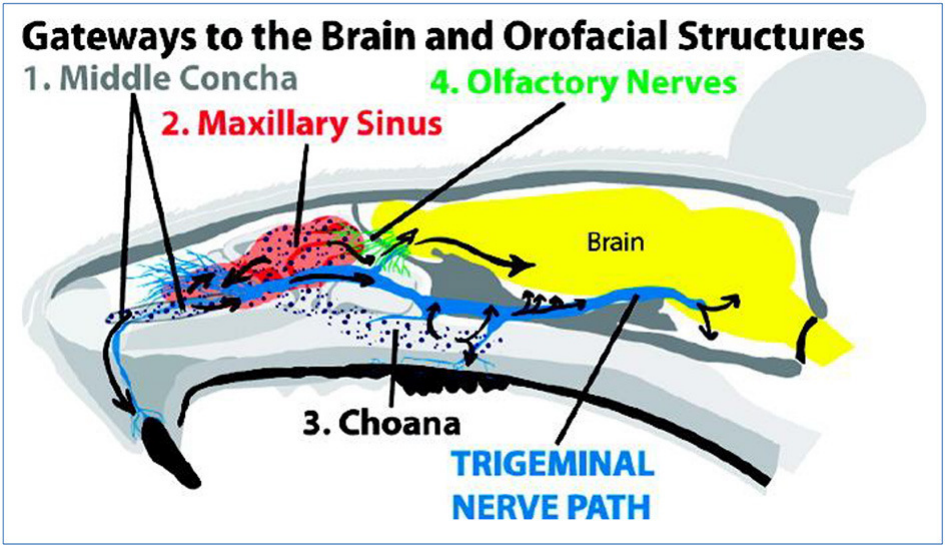
Intranasal administration delivers IRdye 800 to the trigeminal nerve. The trigeminal nerve (see inset) has high IRdye 800 concentrations in four locations: (1) the maxillary incisal nerve as it passes through the middle concha, (2) the infraorbital nerve as it passes through the maxillary sinus, (3) the septal branch, and (4) maxillary molar branch as they pass through the choana. The maxillary teeth and trigeminal nerve receive IRdye based on its proximity from these trigeminally connected structures. Reprinted with permission from ref. Johnson et al. (2010). Copyright (2010) American Chemical Society.

The trigeminal pathway is another important route for delivery of therapeutic agents to the brain. The trigeminal nerve with three branches, including the ophthalmic nerve, maxillary nerve, and mandibular nerve, control the respiratory region of the nasal cavity and sensation of the nasal cavity. Among them, ophthalmic and maxillary nerves bring the information from the nasal cavity to the CNS by controlling the nasal mucosa. So, numerous drug delivery systems for the brain or nerves usually use these two branches as a target for the delivery of drugs (Johnson et al., 2010; Venereau et al., 2016). Drugs enter the brainstem through pons by the trigeminal nerve controlling the nasal cavity and then travel to caudal and rostral parts of brain so that transport of drugs to the brain is achieved. Because not only the olfactory pathway but also the trigeminal pathway could deliver drugs to the rostal area of the brain, it difficult to distinguish when drugs are intranasally administered to the brain by nasal administration (Thorne et al., 2004; Johnson et al., 2010) as shown in Figure 2.

Cerebrospinal fluid (CSF) in the subarachnoid space and nasal lymphatics provide a pathway for therapeutics to both CSF and other areas of the CNS (Dhuria et al., 2010). Some radiolabeled tracers were injected into the CSF in cerebral ventricles or subarachnoid space drain to olfactory bulbs, which then traveled into channels and entered the nasal lymphatic system and cervical lymph nodes (Johnston et al., 2004), which demonstrated the reach of drugs to the CNS after intranasal drugs moved from the nasal cavity to the CSF, the brain interstitial spaces, and perivascular spaces. Moreover, the distribution of neurotherapeutics in CSF also proves the mechanism of nose-to-brain drugs (During et al., 2003).

### Mechanism of Drug Nasal Delivery and Absorption

The principal process in the delivery and absorption of a drug by the nasal cavity route is through the mucus. Mucin, which is formed from mucus, is a protein that has the potential to bind with solutes and thus affect the diffusion process. There are numerous mechanisms for nasal delivery and absorption through the mucosa, including paracellular and transcellular routes (Duvvuri et al., 2003). Paracellular transport is an inverse correlation between intranasal absorption and the molecular weight of water-soluble compounds. When a molecular weight is >1,000 Daltons, drugs showed poor bioavailability (Huang et al., 1985; Alex et al., 2014; Li Y. et al., 2018). The second mechanism is a transcellular process and is responsible for the transport of lipophilic drugs that show a rate dependency on their lipophilicity (Kangmieler et al., 2014; Proschak et al., 2017). As discussed above, to enhance bioavailability, permeation enhancers are necessary (Wang and Chow, 2014; Yamamoto et al., 2017). Permeation enhancers would induce reversible modifications on the structure of the epithelial layer by modifying the phospholipid bilayer (Dhuria et al., 2010; Mouez et al., 2014).

### Advantages and Limitations of Nasal Drug Delivery Route

An intranasal drug delivery system offers a non-invasive, effective, reliable, direct, and alternative route to the CNS *via* the neural connections between the nasal mucosa and the brain (Graff and Pollack, 2005), which is one of the most permeable and highly vascularized sited for drug administration and the onset for therapeutic action (Illum, 2000; Thorne and Frey, 2001). Some distinctive advantages for drug delivery are as follows: (Fortuna et al., 2014; Kumar et al., 2016; Kashyap and Shukla, 2019; Nidhi et al., 2019; Kaneko et al., 2020; Wang et al., 2020) (1) A large surface for drug absorption; (2) Convenience and good patient compliance; (3) Rapid attainment of therapeutic drug levels in the blood; (4) High drug permeability, especially for lipophilic and low molecular weight drugs; (5) Avoidance of harsh environmental and gastrointestinal conditions; (6) Bypassing of the hepatic first-pass metabolism; (7) Potential direct drug delivery to the brain along the olfactory nerves; (8) Direct contact site for vaccines with lymphatic tissues; (9) Convenient for patients in long-term therapy; and (10) An alternative route for drugs with poor stability in fluids. In the nasal epithelium, the therapeutic agents are delivered to the olfactory bulb and brainstem, and disperse to other CNS areas *via* pulsatile flow within the perivascular spaces of cerebral blood vessels which contributes to drug absorption. It is almost equal to intravenous injections in some instances, owing to the unique direct connection between the brain and the nasal cavity (Angeli et al., 2019; Musumeci and Bonaccorso, 2019; Craft et al., 2020). Intranasal administration is the only route that connects the brain with the outside environment (Mistry et al., 2009), which has received attention due to its wide drug delivery potential, including nucleotides, peptides, proteins, and even stem cells. Moreover, the nasal route of drug delivery includes both local and systemic drug delivery. A wide range of pharmaceutical dosage forms, including solutions, gels, suspensions, emulsions, liposomes, and microparticles administered *via* nasal route (Jullaphant et al., 2009; Kitiyodom et al., 2019; Lengyel et al., 2019; Salade et al., 2019), achieved an enhanced targeting ability and reduced systemic side-effects.

**Figure 3:**
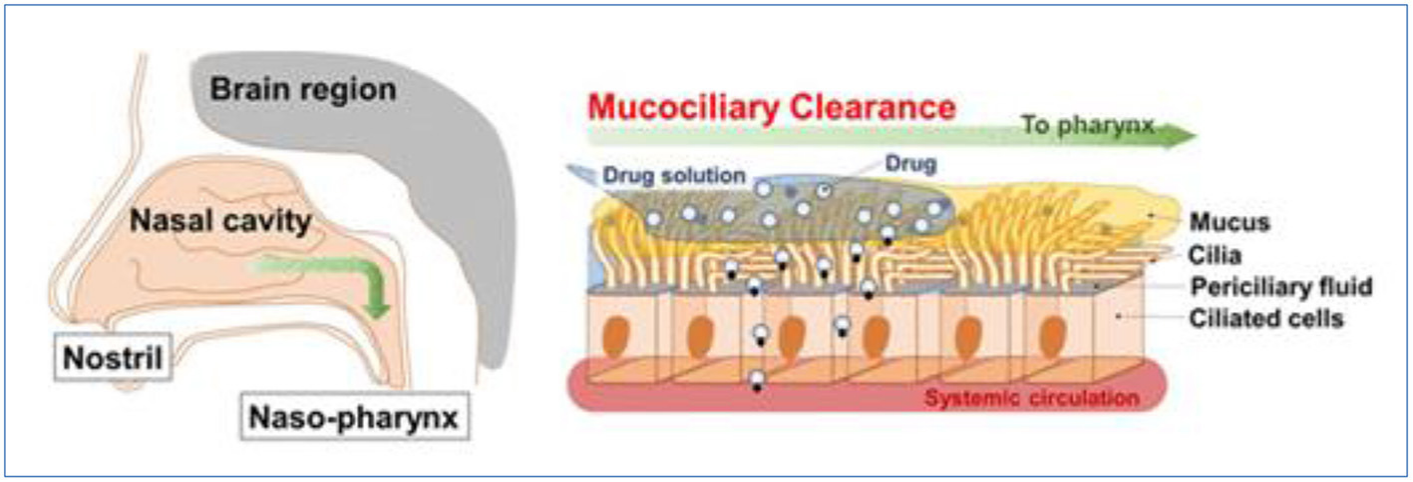
Nasal mucociliary absorption route after intranasal administration, reprinted with permission from Inoue et al. (2018). Copyright (2018) American Chemical Society.

However, some vital factors and physicochemical properties influence the nasal absorption of drugs: (1) Physiochemical properties of the drug, including molecular weight, particle size, lipophilic-hydrophilic balance, and enzymatic degradation; (2) Nasal effect, including membrane permeability, environmental pH, mucociliary clearance, etc.; and (3) Delivery effect, including formulation, drugs distribution and deposition, and viscosity of the formulation (Alagusundaram et al., 2010; Chaturvedi et al., 2011; Bakri et al., 2018; Inoue et al., 2020). For example, some polar drugs or macromolecules are not absorbed in high enough concentrations for their poor membrane permeability, rapid clearance, and enzymatic degradation within the nasal cavity. And the nasal mucosa and physiological and anatomical factors, including nasal blood flow, enzymatic degradation, mucociliary clearance, the physical condition of the nose, including nasal atrophic rhinitis and severe vasomotor rhinitis, could also reduce the capacity of nasal drug absorption and bioavailability as Figure 3 (Sintov et al., 2010; Qian et al., 2014; Mansuri et al., 2016; Rohm et al., 2017; Inoue et al., 2018; Rohrer et al., 2018; Akel et al., 2020; Badhe and Nipate, 2020). These limitations must be addressed in the design of drug absorption by the nasal route.

## Nasal Drug Delivery Devices

It should be noted that efficient and novel nasal drug delivery devices used for direct transport of drugs from nose to brain is another important strategy for the improvement of diagnosis and treatment effect, which help drugs to be transported to the brain *via* the olfactory/trigeminal pathway. Some devices, including droppers, syringes, pressurized meter dose inhalers, Breathe Powered Bi-directional nasal devices, and pressurized olfactory delivery devices, were adopted in clinical treatment and were also categorized into devices with liquid, powder, and semisolid formulations (Djupesland, 2013; Erdő et al., 2018). The right delivery system depended on the type of drug formulation. Powder formulations with high stability often stick to the nasal mucosa before it is cleared. Liquid formulations are the oldest, cheapest, and simplest method. Popular nasal sprays spread easily and deposit in the olfactory region. The spray would distribute in the nasal cavity through nasal mucociliary clearance. But the limitation of nasal drops or spray devices depends on the self-administration technique.

### Powder Devices

**Figure 4:**
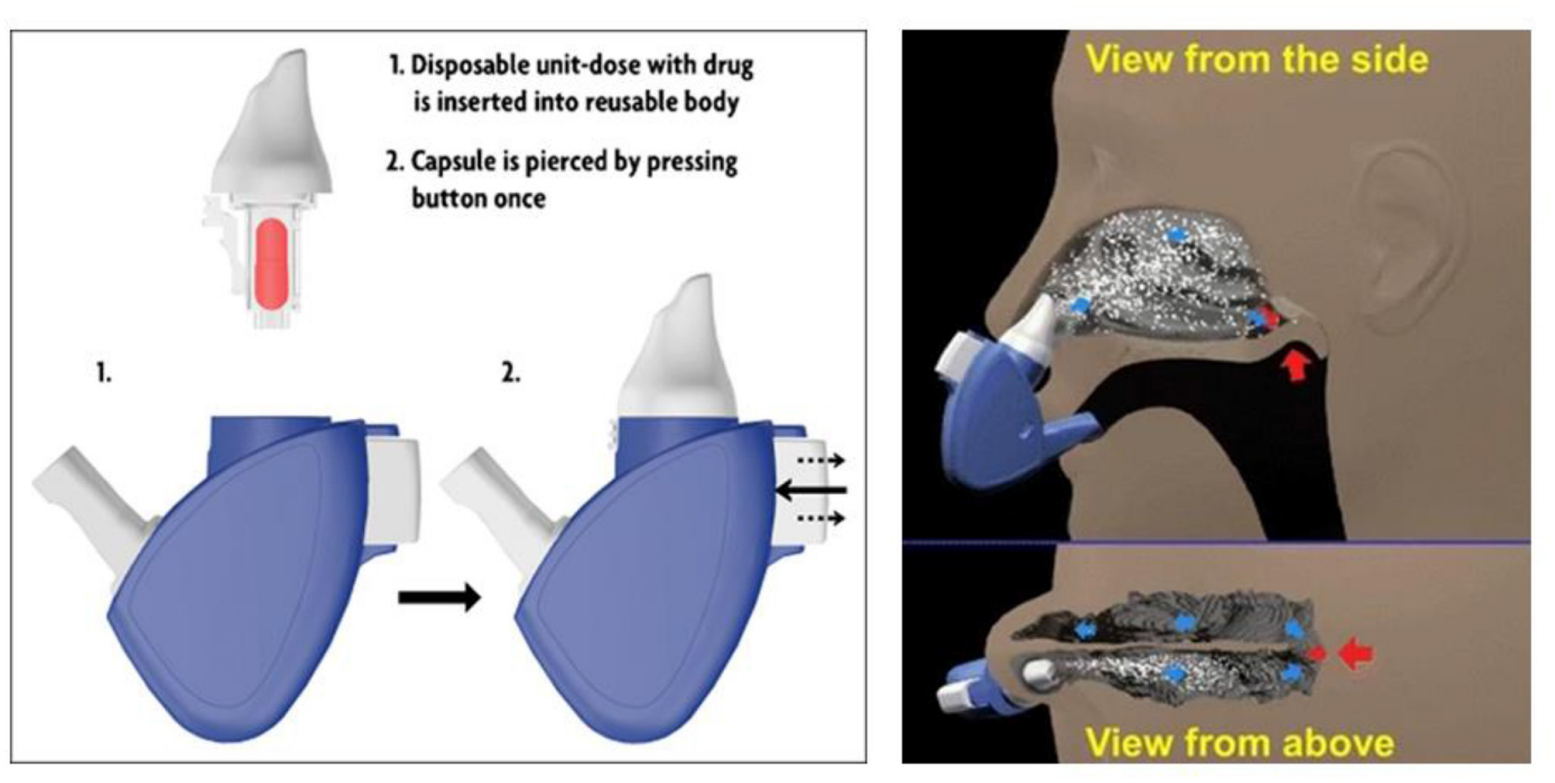
Schematic illustration of the breath-powered Bi-Directional™ technology. Reprinted with permission from Djupesland (2013). Copyright (2013) Springer Nature.

Compared with liquid (solution or suspension) and gel formulations of drugs, powder particles are stable and not easily dissolved so they can remain in the nasal mucosa for a long time. Moreover, because of free preservatives, powder dosages could be administrated in a large dose and prevent microbial contamination. Deposition and absorption of a powder formulation of drugs for nasal delivery depend on many factors, including size and shape of powder particle, flow characteristics, and solubility (Djupesland, 2013) as shown in Figure 4.

An insufflator composed of straw or tube with drugs could directly deliver drugs to the olfactory region, although usually local anesthetics or decongestants are needed before insufflations delivery. Direct Haler, designed by a Danish company, delivered fine particles into the nasal cavity with lungs exposure. Compared with pressurized metered dose inhalers (pMDI) with poor patient acceptability, insufflators utilized the exhalation force of patients with normal temperatures and was popular. The Direct Haler device exhibited excellent advantages such as being free from contamination and preservatives, priming, and cleaning. Because the Direct Haler's route of drug delivery is from nose to brain, targeting the nasal valve is necessary. Smaller powder particles below 5 μ are used for olfactory absorption to increase the drug deposition to lungs. Bi-Directional technology uses the normal breath processes of the body to deliver drugs in liquid or powder form on the nasal epithelium (Djupesland, 2013). The nasal valve is a barrier for delivery of drugs because the nasal cavity is narrow. Optinose, a biopharmaceutical company, proposed closed-palate Bi-Directional™ Breath Powered® DDSs, which used an exhale device to target the drug beyond this valve (Djupesland, 2013; Djupesland et al., 2014). As we know, oxytocin has already been used for treatment of patients on the autism spectrum, however, a higher dose would lead to adverse effects. Quintana et al. (2015) proposed a randomized four-way crossover trial using a breath powered optinose device to study the relationship between the effect of oxytocin on social cognition with dose. Through this device, a lower dose was observed with a breath powered optinose device to deliver oxytocin across the nasal mucosa to the brain and demonstrated that lower doses are more efficacious than higher in producing a cognitive response.

Dry powder inhalers (DPI), which contain drug particles suspended or dissolved in solvent when they contact the nasal mucosa, deliver small doses to the nasal cavity. Teijin Puvlizer Rhinocort® is the earliest dry powder inhaler on the market. Rhinocort Turbohaler®, Rhinicort Puvlizer®, and Erizas® are three popular nasal dry inhalers used for the treatment of rhinitis (Gomes dos Reis et al., 2020). Dry powder inhalers are simple in design, cheap, can be operated without medical supervision, and doses range from μg to mg.

Bespak developed Unidose-DP™, resembling Flit Lizer technology, which was composed of a sealed container used for delivering a single shot of a drug. About 95% of the drug was delivered to the nasal cavity and about 60–70% reached the nasal vestibular region (Kaye et al., 2009). SoluVent™ is a powder delivery device and forces the powder to the nasal cavity. Vaccines have been delivered through this device (Huang et al., 2004). μco™ System was designed by Shin Nippon Biomedical Laboratories for drug absorption and decreased dose requirement. Powder carriers with systemic delivery, low molecular weight, and high molecular weight enhanced safety in clinical treatment (Bell et al., 1971; Ugwoke et al., 1999). μco™ System was designed by Shin Nippon Biomedical Laboratories. The μco™ carrier technology was based on a mucoadhesive powder drug carrier, which increased drug absorption and decreased doses of drug and provided a high activity and long contact time (Djupesland, 2013).

### Liquid-Based Devices

Catheter-delivered drugs are the simplest method of drug delivery to the nasal cavity through the insertion of a catheter in the nose cavity. This technique is usually accompanied with anesthesia or a sedative to deliver the liquid. Because mucosa is sensitive to the deposition site, it is a major issue with this method of drug delivery. By blowing with the mouth, solution filled in the catheter enters into the nostril by the other end of the catheter in the nose cavity (Trangsrud et al., 2002; Berger et al., 2007; Djupesland, 2013).

Drops are another important method of nasal drug delivery for liquid formulations and have been used for decades due to their cost effectiveness and easy manufacture. However, this method risks microbial contamination and chemical instability. Glass droppers are a typical single dose drug delivery, and spray pumps would obtain a multidose administration. However, spray pumps are expensive and often are replaced by cheap disposable pipettes in clinical treatment. Pipettes, especially in treatment for nasal decongestion and irrigation purposes, often replace the drop or spray pump in the European market (Penttilä et al., 2000).

The olfactory epithelium is located in the upper part of the nose and is about 3–10% of the surface area of the nasal cavity between the nose and brain. It is a challenge to deliver therapeutic molecules *via* the nasal cavity due to its turbinate restriction (Morrison and Costanzo, 1990; Fonseca-Santos et al., 2015; Brozzetti et al., 2020). The pressurized olfactory device (POD) of Impel NeuroPharma (Seattle, Washington), containing a suitable tank, compressed air or nitrogen, chlorofluorocarbon (CFC) or hydrofluoroalkane (HFA), used as a propellant and air chamber, was realized to target the drug to the olfactory part of the nasal cavity. POD could transport both liquid and powder formulations through the nasal epithelium. Impel NeuroPharma deliver aqueous and powder forms of cholinergic reactivator (pralidoxime, 2-PAM) by POD with a higher concentration of 2-PAM in the brain (Brunelle et al., 2012; Bachurin et al., 2018).

## Intranasal Delivery Carriers of Antidepressants In Clinical Studies

Conventionally, drugs that are administered through the intranasal route are in the forms of solutions, suspensions, gels, emulsions, and powders. However, these conventional dosage forms meet many problems, such as a lack of dose precision, high particle size, high viscosity, and lack of drug stability. To enhance patient convenience, adherence, and comfort, alternating drug formulations and routes of administration should be used. As we know, oral and injectable routes of drug delivery result in a relatively well-tolerated therapeutic management, but it isn‘t feasible in all patients. The pharmacokinetic and physicochemical properties of drugs used in intranasal administration and drug properties for antidepressant medications are summarized in Table 3 (Mathias and Hussain, 2010; Bento et al., 2014). Two important factors, degree of lipophilicity (log P) and absorption of the acid dissociation constant (pKa), would most affect drug intranasal administration. Theoretically, based on these chemical properties, alternative routes of administration for antidepressants depending on different and novel formulations, including nanogels, nanostructured lipid, liposomes nanoparticles, and nanoemulsions/microemulsion, could be explored as drug delivery systems for intranasal delivery (Joshi et al., 2006; Wong et al., 2017; Ding et al., 2019a).

**Table 3:** Physicochemical properties of intranasal antidepressants.

**Drug**	**Adult oral dose**	**Molecular weight of parent molecule**	**Log *P***	**Basic pKa**
Citalopram	20–40 mg	324.4	3.72	9.57
Desvenlafaxine	50–100 mg	263.4	2.80	9.33
Doxepin	75–300 mg	279.4	3.91	9.19
Escitalopram	10–20 mg	324.4	3.72	9.57
Fluvoxamine	50–300 mg	318.3	2.55	9.39
Mirtazapine	15–45 mg	265.4	3.09	8.1
Paroxetine	20–50 mg	329.4	3.23	9.68
Selegiline	5–30 mg	187.3	3.79	7.53
Trazodone	50–400 mg	371.9	2.42	7.52
Venlafaxine	75–375 mg	277.4	3.02	9.27

### Nanogels

Intranasal delivery, as an attractive alternative method, has been used in the treatment of major depressive disorder, social anxiety disorder, generalized anxiety disorder, and panic disorder. However, low residence time of drugs in the nasal cavity would affect absorption and in turn bioavailability of the drug, which has been a crucial problem that must be faced in clinical treatment. That is, the anatomic and physiologic characteristics of nasal mucosa must be considered in the design of nasal dosage forms used for solving the rapid mucocilliary clearance (MCC) (Grinberg et al., 2017; Patel and Patel, 2017; Li S. et al., 2018; Jiang et al., 2019; Qiu et al., 2020). Gel as a nasal mucoadhesive has been very attractive due to its fluid-like qualities prior to nasal administration and can easily be administered by drops to allow for accurate drug dosing. Moreover, gelation can be constructed by using thermos-sensitive smart polymers which use sensing with nasal temperature (Dias et al., 2010; Singh et al., 2013; Naik and Nair, 2014; Gholizadeh et al., 2019; Zhang Y. et al., 2019). Bhandwalkar and Avachat (2013) reported an *in situ* mucoadhesive thermoreversible gel used for improving bioavailability of the antidepressant drug, venlafaxine hydrochloride, composed of Lutrol F127 (18%). Mucoadhesive thermoreversible *in situ* nasal gel enhanced nasal residence time and absorption of venlafaxine hydrochloride across the nasal mucosal membrane to improve bioavailability of the drug as compared with the oral route and administration of an equivalent dose. A thermoreversible biogel system based on chitosan and glycerophosphate for delivery of doxepin to the brain through intranasal administration was also reported (Naik and Nair, 2014). The antidepressant doxepin hydrochloride was doped into the mixture of chitosan and glycerophosphate with polyethylene glycol. The mixture gelled rapidly at 37°C, and returned to sol state upon cooling. The gels showed potential as effective drug delivery methods to the brain via the nose. Moreover, an intranasal chitosan nanoparticles (NPs)-loaded *in situ* gel, Tramadol HCl, against depression was developed (Kaur et al., 2014). Chitosan nanoparticles (NPs) were prepared by ionic gelation method followed by the addition of developed NPs within the Pluronic and HPMC-based mucoadhesive thermo-reversible gel. Depression induction of forced swim test and various behavioral and biochemical parameters demonstrated that intranasal TRM HCl NP-loaded *in situ* gels were a promising formulation for the treatment of depression.

### Nanostructured Lipid

Nanostructured lipid carriers (NLCs), which are the nanostructured particles produced from the two-phased blend of solid and liquid lipids, have gained increasing attention during the last years. A characteristic feature of the NLC is its controlled “imperfections” nanostructure, which is created by lipid particle matrix as imperfectly as possible by spatially very different molecules being mixed into them. The imperfections in NLC increase drug loading capacity and minimize or avoid drug loss resulted from light,oxidation and hydrolysis. NLC has been used as a delivery carrier of therapeutic substances to the brain (Muchow et al., 2008).

Duloxetine (DLX) is the first class of anti-depressants which ensures rapid and sustained efficacy in treatment of both emotional and physical symptoms of depression. On oral administration of DLX, its bioavailability, kept about 50%, undergoes hepatic first pass metabolism (Martin et al., 2003). Moreover, as we know, the efficacy of antidepressants relies upon their continued presence at the site of action (brain), however, as DLX is an oral as well as intravenous administration, the BBB would restrict the access of antidepressant drugs to the brain, leading to the reduction in dose and side effects (Laffleur and Keckeis, 2020). Alam et al. (2014) reported a nanostructured lipid carrier system of intranasal administration in which the antidepressant drug, duloxetine (DLX), was contained to circumvent the BBB and maintain prolonged release at the site. DLX-NLC was prepared by mixing melted solid lipid (glyceryl monostearate) and liquid lipid (capryol PGMC), after which both of them were homogenized with hot surfactants aqueous solution (80°C pluronic F-68, 1.5%; sodium taurocholate, 0.5%) and lyophilized. The biodistribution studies suggested that DLX-NLC formulation had a better brain targeting efficiency and higher concentration of DLX in the brain by intranasal administration compared with the intravenous administration in the treatment of depression.

### Liposomes

To date, two of the most popular pathways to delivery drugs via nasal administration that are also highly lipid-soluble compounds are hydrophilic and semilipophilic substances. However, highly lipid-soluble compounds usually require several steps and are relatively slow. Hydrophilic and semilipophilic substances provide a relatively quick absorption into the cerebrospinal fluid (CSF). Now liposomes have been used as an effective delivery system to the brain, due to particles being entrapped into the compounds and preventing the rapid degradation through the BBB and distribution in the brain tissue (Talegaonkar and Mishra, 2004; Krauze et al., 2006; Ding et al., 2019b). Liposomes could be prepared into nanosize vesicles (20–100 nm) by phospholipids and similar amphipathic lipids. Lipid bilayers of liposomes are similar to membranes in living cells; thus, they can carry lipophilic substances such as drugs within these layers as cell membranes. Specially, because the drug is entrapped in liposomes, a decreased dose of a compound is usually expected (Sharma and Sharma, 1997; Druliskawa and Dorotkiewiczjach, 2010; Loirapastoriza et al., 2014).

Quercetin, 3,5,7,3′,4′-pentahydroxylflavone, as a bioflavonol, usually found in foods such as onions, apples, tea, and red wines, could exert beneficial actions on the CNS, such as being antianxiety. To apply quercetin for the treatment of antidepressants, some limitations of quercetin must be solved, such as its poor absorption and very low distribution to the brain after oral administration (Dandrea, 2015; Wang et al., 2016) for rapid metabolism and bypassing of the BBB. The nasal route of quercetin can serve as a potential pathway for systemic drug delivery because of the high degree of vascularization and permeability of the nasal mucosa. Priprem et al. (2008) developed intranasal quercetin liposomes and assessed the anxiolytic and cognitive effects on rats based on lipid thin film formation and extrusion. The lipid thin film was a mixture of egg phosphatidylcholine (EPC) and cholesterol (chol). Due to the semilipophilic character of quercetin, it was mixed to the EPC/chol using a vehicle (water, 50% ethanol, and 50% PEG). Quercetin liposomes showed anxiolytic and cognitive-enhancing effects. Moreover, a lower dose and a faster rate were observed with intranasal quercetin liposomes which exhibited that the intranasal quercetin liposomes were effective in the delivery of quercetin to the central nervous system. Piperine, which encapsulated liposomes for intranasal delivery, was investigated in male Wistar rats (Priprem et al., 2011). Piperine, as a major alkaloid of black long pepper, had been proven to possess various activities, including anti-inflammatory and anti-depressant (Lee et al., 2005; Menneson et al., 2020). A lipid film of EPC and chol was used in the presence of piperine and chloroform. Subsequently, piperine-encapsulated liposomes were prepared. Intranasal liposomes also proved their potential in the delivery of piperine, at a low dose, to exert its antidepressant and cognitive enhancing activities.

### Nanoparticles

Among intranasal drug delivery systems, the mucoadhesive chitosan nanoparticles (NPs) possess the ability to reduce the mucociliary clearance, pass through the tight junctions of cells transiently, and provide a drug transport route from the nasal membrane to the brain by the paracellular route because of its particle size, enhanced permeability, and ability to encapsulate various ingredients (Maculotti et al., 2005; Vllasaliu et al., 2010; Chen J. et al., 2020). Venlafaxine (VLF) is a dual action antidepressant [serotonin and norepinephrine reuptake inhibitors (SNRI)]. Oral therapy of VLF meets some problems, such as slow onset of action and side effects like tachycardia, increased blood pressure, fatigue, headache, dizziness, sexual dysfunction, and low bioavailability (40–45%).

Haque et al. (2012) prepared venlafaxine (VLF)-loaded chitosan nanoparticles (NPs) to enhance the uptake of VLF to the brain *via* intranasal delivery. One phase which contained a solution of polycation chitosan mixed with another that contained a solution of polyanion sodium tripolyphosphate (TPP) at room temperature and VLF was dissolved into chitosan solution at the drug polymer ratio of 1:1 before the addition of TPP. The brain/blood ratios of VLF for VLF (i.v.), VLF (i.n.), and VLF chitosan NPs (i.n.), were 0.0293, 0.0700, and 0.1612, respectively, at 0.5 h, which indicated a better brain uptake of VLF-chitosan NPs, higher drug transport efficiency (508.59), and direct transport percentage (80.34) of VLF chitosan NPs. In another piece of research, Haque et al. (2014) investigated Venlafaxine-loaded alginate chitosan nanoparticles (VLF AG-NPs) for the treatment of depression via intranasal (i.n.) nose to brain delivery route. Pharmacodynamic studies of the VLF AG-NPs for antidepressant activity were carried out *in-vivo* by forced swimming test, which indicated that VLF AG-NPs (i.n.) treatment significantly improved the behavioral analysis parameters including swimming, climbing, and immobility. Tong et al. (2017) prepared Desvenlafaxine-loaded PLGA-chitosan nanoparticles by solvent emulsion evaporation technique and the optimized Desvenlafaxine-loaded PLGA-chitosan nanoparticles based on intranasal administration significantly reduced the symptoms of depression. Intranasal Desvenlafaxine PLGA chitosan nanoparticles also enhanced the Desvenlafaxine in brain together with their brain/blood ratio at different time points, which demonstrated its better efficacy in treatment of depression. Singh et al. (2015) presented the study of thiolated chitosan nanoparticles (TCNs) which were synthesized by the ionic gelation method, to enhance the nasal delivery of selegiline hydrochloride for effective treatment of depression. In the evaluation of drugs on animals, selegiline hydrochloride—TCNs successfully attenuated oxidative stress and restored the activity of the mitochondrial complex. In an evaluation of behavioral parameters, TCNs successfully restored the impaired locomotor activity and normal sucrose consumption was found on treatment. TCNs seemed to be promising carriers for nose-to-brain delivery in the evaluation of antidepressant activity.

### Nanoemulsions /Microemulsion

Intranasal delivery of drugs allows the drug to directly enter the brain by bypassing the BBB and avoids extensive hepatic and intestinal metabolism. This route has been a convenient and reliable route. Several new formulations are used to deliver drugs to the brain by olfactory, neuronal, and trigeminal pathways. Nanoemulsions are a promising and novel formulation to deliver lipophilic drugs to the brain through the intranasal route, which is an optically isotropic and thermodynamically stable system composed of oil, water, and surfactant (and/or co surfactant) (Mehta et al., 2008). Due to its high solubilization of lipophilic drugs, stability, ease of preparation, and handled stabilization of hydrolytically susceptible compounds, it provided the advantages of bioacceptability, biodegradability, and rapid uptake by the brain (Abouhussein et al., 2018; Pires and Santos, 2018). Many drugs, including Sumatriptan (Vyas et al., 2006a; Ganger and Schindowski, 2018), Zolmitriptan (Vyas et al., 2005; Islam et al., 2020), Cabergoline (Sharma et al., 2009; Pires and Santos, 2018), Clonazepam (Vyas et al., 2006b; Costa et al., 2019), Nimodipine (Zhang et al., 2004), Tacrine (Jogani et al., 2008), and Diazepam (Li et al., 2002), have been administered *via* the nasal route in the form of microemulsion (Mason et al., 2006; Jones et al., 2017; Singh et al., 2017; Sindhu et al., 2018). Pandey et al. (2016) developed a paroxetine-loaded nanoemulsion (o/w type) for direct nose-to-brain delivery. Nanoemulsions were constructed by the spontaneous emulsification technique using oil phase, including Capmul MCM, Solutol HS 15, and propylene glycol, surfactant and co-surfactant, for a delivery system via the nasal route to treat depression. Intranasal treatment of depressed rats with paroxetine nanoemulsion significantly improved the behavioral activities in comparison to oral paroxetine control groups. Biochemical estimation results suggested that the paroxetine-loaded nanoemulsion was effective in enhancing the depressed levels of glutathione and decreasing the elevated levels of thiobarbituric acid reactive substances (TBARS). Thakkar et al. (2013) prepared mirtazapine microemulsion for intranasal delivery microemulsions (MME-26% w/w, mirtazapine) through completely dissolving mirtazapine in a mixture of oil Capmul MCM (O-7% w/w), surfactant Tween-80 (S-33.75% w/w), co-surfactant, and Polyethylene Glycol 400 (CoS-11.25% w/w). Pharmacokinetics, swim tests, locomotor activity, maze tests, and brain/blood uptake ratios all demonstrated a more rapid and a larger extent of transport of mirtazapine into the brain with intranasal mirtazapine mucoadhesive microemulsion.

## Conclusions And Perspectives

Direct intranasal drug transportation to the brain has been highlighted as a potential strategy for addressing antidepressant therapy, because it would break through the bottleneck of the blood-brain barrier and enhance targeting ability. It is well-known that the progress of intranasal drug transportation benefits from nanocarrier-based formulations, however, although encouraging and tremendous developments have achieved, to explore and apply all kinds of drug carriers for antidepressant treatment by the intranasal route in clinics retains many challenges. Drugs in the intranasal delivery route are more sensitive for enzyme, acid, and hepatic metabolism than other routes. Moreover, the absorption and bioavailability of drugs can usually be influenced by rapid drug elimination, limited administration volume, and so on. In addition, the use of intranasal drugs is still at an early stage and far from clinical practice. As we know, esketamine nasal spray was recently approved as the first intranasal prescription medicine used for the treatment of depression. These findings corroborate that nose-to-brain drug delivery could provide a strong effective solution for treatment-resistant depression. Regulatory issues in safety and quality aspects need to be satisfied to translate these methods from research to the market. The pharmaceutical industry must be evolving and improving processes for manufacturing accurate and repeatable intranasal dose in the coming years. In this review, we introduced the absorption mechanism, advantages including large absorption surface area, good patient compliance, rapid and high drug permeability, bypassing of hepatic first-pass metabolism, potential direct drug delivery to the brain along the olfactory nerves, and limitations, including rapid drug elimination prompted by mucociliary clearance (5 mm/min for healthy humans), limited administration volume (25–200 ml), and the presence of enzymes that degrade in nasal drug delivery route (Lechanteur et al., 2017, 2019; Chen S. et al., 2020; Lechanteur and Evrard, 2020; Sabir et al., 2020; Taipaleenmaki and Stadler, 2020). Then, we systematically summarized the development of intranasal delivery carriers of antidepressants in clinical studies including nanogels, nanostructured lipid, liposomes nanoparticles, and nanoemulsions/microemulsion. Finally, recent progress, challenges, and future perspectives of intranasal delivery carriers in antidepressant treatment were discussed.

## Author Contributions

JX: conceptualization and writing—original draft preparation. JT: investigation and writing—original draft preparation. JW: supervision, writing—reviewing, and editing. All authors read and approved the final manuscript.

## Conflict of Interest

The authors declare that the research was conducted in the absence of any commercial or financial relationships that could be construed as a potential conflict of interest.
